# Identification of CD166 as a Surface Marker for Enriching Prostate Stem/Progenitor and Cancer Initiating Cells

**DOI:** 10.1371/journal.pone.0042564

**Published:** 2012-08-03

**Authors:** Jing Jiao, Antreas Hindoyan, Shunyou Wang, Linh M. Tran, Andrew S. Goldstein, Devon Lawson, Donghui Chen, Yunfeng Li, Changyong Guo, Baohui Zhang, Ladan Fazli, Martin Gleave, Owen N. Witte, Isla P. Garraway, Hong Wu

**Affiliations:** 1 Department of Molecular and Medical Pharmacology, University of California Los Angeles, Los Angeles, California, United States of America; 2 Institute for Molecular Medicine, University of California Los Angeles, Los Angeles, California, United States of America; 3 Howard Hughes Medical Institute, University of California Los Angeles, Los Angeles, California, United States of America; 4 Department of Microbiology and Molecular Genetics, University of California Los Angeles, Los Angeles, California, United States of America; 5 Department of Urology, University of California Los Angeles, Los Angeles, California, United States of America; 6 The Vancouver Prostate Centre and University of British Columbia, Vancouver, British Columbia, Canada; 7 Eli and Edythe Broad Center of Regenerative Medicine and Stem Cell Research, University of California Los Angeles, Los Angeles, California, United States of America; The University of Texas M.D. Anderson Cancer Center, United States of America

## Abstract

New therapies for late stage and castration resistant prostate cancer (CRPC) depend on defining unique properties and pathways of cell sub-populations capable of sustaining the net growth of the cancer. One of the best enrichment schemes for isolating the putative stem/progenitor cell from the murine prostate gland is Lin^-^;Sca1^+^;CD49f^hi^ (LSC^hi^), which results in a more than 10-fold enrichment for *in vitro* sphere-forming activity. We have shown previously that the LSC^hi^ subpopulation is both necessary and sufficient for cancer initiation in the *Pten*-null prostate cancer model. To further improve this enrichment scheme, we searched for cell surface molecules upregulated upon castration of murine prostate and identified CD166 as a candidate gene. CD166 encodes a cell surface molecule that can further enrich sphere-forming activity of WT LSC^hi^ and *Pten* null LSC^hi^. Importantly, CD166 could enrich sphere-forming ability of benign primary human prostate cells *in vitro* and induce the formation of tubule-like structures *in vivo*. CD166 expression is upregulated in human prostate cancers, especially CRPC samples. Although genetic deletion of murine CD166 in the *Pten* null prostate cancer model does not interfere with sphere formation or block prostate cancer progression and CRPC development, the presence of CD166 on prostate stem/progenitors and castration resistant sub-populations suggest that it is a cell surface molecule with the potential for targeted delivery of human prostate cancer therapeutics.

## Introduction

Despite advances in the early detection and management of prostate cancer, castration resistant prostate cancer (CRPC) remains the second most common cause of male mortality in the United States [1]. Mounting evidence suggests that a subpopulation of prostate cells can initiate prostate cancer and may be responsible for the castration resistance [2], [3], [4], [5]. Therefore, these cancer initiating cells [6] may serve as promising cellular targets for prostate cancer and identification of this subpopulation has become the necessary step toward future effective therapy.

The origins of prostate cancer initiating cells are controversial [7], [8]. Normal prostate from human or mouse contains three different types of cells, namely luminal secretory, basal and neuroendocrine cells. Since human prostate cancer is characterized by loss of basal cells and expansion of luminal cells, several animal models posit that luminal-specific progenitors are the sources of prostate cancer initiation [9], [10], [11]. However, using the tissue regeneration approach, basal cells have proved to be more efficient oncogenic targets for both human and mouse prostate cancer initiation [12], [13]. Interestingly, Xin’s group demonstrated that adult murine prostate basal and luminal cells are self-sustained lineages that can both serve as oncogenic targets for prostate cancer initiation [14].

PTEN plays an important role in human prostate cancer and CRPC development [15] and is inactivated in 20% of primary and 60% of metastatic lesions [16]. The murine *Pten* prostate cancer model (*Pb-Cre^+^;Pten^L/L^*) recapitulates the disease progression seen in humans, including CRPC [17], [18], [19], [20], and shares many signature genetic changes with human disease [17]. Importantly, the *Pb-Cre^+^;Pten^L/L^* model provides a unique tool for studying tumor initiating cells as the majority of luminal cells and subpopulations of basal cells have *Pten* deletion [17], [18]. Using this model, we demonstrated that *Pten* deletion causes an expansion of basal and transient amplifying subpopulations and subsequent tumor initiation *in vivo* [18]. We further showed Lin^-^Sca-1^+^CD49f^hi^ (LSC^hi^) prostate stem/progenitor cells from the *Pten* null prostate are capable of initiating a cancerous phenotype that mimics the primary cancer in the *Pten* null prostate model [19].

Here, we report the identification of a cell surface marker, CD166 or Activated Leukocyte Cell Adhesion Molecule (CD166/ALCAM) that is highly upregulated in human and murine CRPC samples. CD166 can be used to enrich for stem/progenitor sphere-forming cells from both WT and *Pten* null mutant mouse prostates. In addition, CD166 can separate LSC^hi^ mouse stem/progenitor cells into CD166^hi^ and CD166^lo^ subpopulations, with the LSC^hi^;CD166^hi^ subpopulation having much higher sphere-forming activity. We further demonstrate that CD166 can be used as an enrichment maker for isolating human prostate sphere-forming cells and tubule-forming cells.

## Results

### CD166 Expression is Upregulated in Murine Castrated Prostatic Epithelium and can be used for Enriching Stem/progenitor Cells

Rodent prostate contains stem-like cells that are enriched in the castrated prostate gland and can undergo more than 15 cycles of involution-regeneration in response to androgen withdrawal and replacement [21]. We reasoned that castration may also lead to upregulation or enrichment of those stem cell surface molecules that can potentially serve as marker for isolating stem/progenitor cells and for targeted drug delivery. We therefore mined publically available databases describing gene expression profiles of murine prostates at day 0 and day 3 post-castration [22], [23]. We focused on those genes that fell in the gene ontology category of ‘plasma membrane’ and identified CD166/ALCAM as one of only two common castration-enriched cell surface molecules (Table S1). CD166 was significantly increased (1-tail t-test <0.015) 3 days after castration as compared to intact mice. While Cxcl12 is also upregulated, we chose not to focus on this gene as it is a chemokine and not amenable for FACS-mediated stem/progenitor cell enrichment.

**Figure 1:**
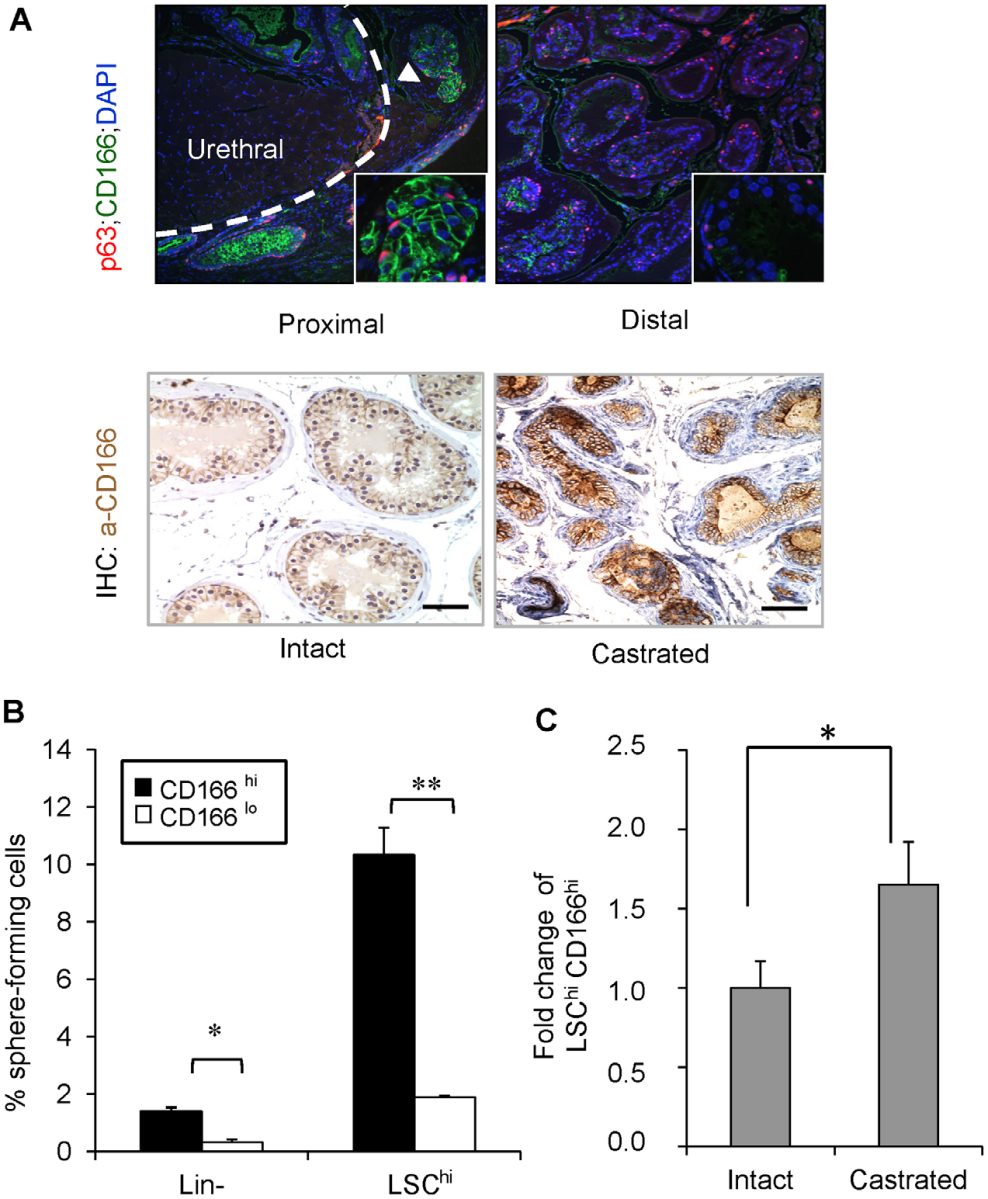
CD166 expression is upregulated in castrated prostate epithelium and CD166 can be used to enrich stem/progenitor cells in WT mice prostate. (A) Top: Comparison of p63 (red) and CD166 (green) co-IF staining between prostate proximal region and distal region. Bottom: IHC for CD166 expression from intact vs. castrated mouse prostate. Scale bar: 50 µm. (B) Lin^-^;CD166^hi^, Lin^-^;CD166^lo^, LSC^hi^;CD166^hi^, and LSC^hi^;CD166^lo^ cells were isolated by FACS from 8- to 12-week-old mice. Graph shows the percentage of sphere-forming cells, based on the spheres from each population per 2500 cells plated after 8 days of growth. Data shown as mean +/− STD (**, p<0.001, n = 3). (C) Fold change of LSC^hi^;CD166^hi^ content based on intact WT from FACS analysis (*, p<0.05, n = 3).

CD166 is a type I transmembrane protein of the Ig superfamily that mediates cell-cell interactions via heterophilic (CD166-CD6) and/or homophilic (CD166-CD166) mechanisms [24], [25]. We found that in the intact mice, CD166 is preferentially expressed in the stem/progenitor-enriched proximal region [21] but low in the stem/progenitor-poor distal region of the WT prostate (Figure 1A upper panels). CD166 protein levels are also up-regulated immediately following castration (Figure 1A lower panels; comparing day 0 and day 3 post- castration).

Prostate stem/progenitor cells are characterized by their ability to form spheres *in vitro* [26]. We performed the sphere-forming assay using sorted CD166^hi^ and CD166^lo^ cells and found that CD166^hi^ cells have significantly higher sphere-forming activity compared to CD166^lo^ cells (Figure 1B, left). Since we had previously developed the LSC^hi^ enrichment scheme [26], which yields 10-fold enrichment of WT sphere-forming cells, we tested whether CD166 can be used for further enriching sphere-forming activity. We gated LSC^hi^ cells according to their CD166 expression and found that LSC^hi^;CD166^hi^ cells have 5-fold higher sphere-forming activity as compared to their LSC^hi^;CD166^lo^ counterpart (Figure 1B, right). Therefore, CD166 can be used as a marker to further enrich sphere forming cells within the WT prostate. Serial passaging of the spheres generated from LSC^hi^;CD166^hi^ cells demonstrated that this enhanced sphere-forming activity could be maintained *in vitro* through at least three passages (Figure S1A). In contrast, less spheres were generated from LSC^hi^;CD166^lo^ cells (P0–P2) and cannot undergo continuous passage due to the limited cell number. We observed no significant difference in the sphere size distribution between LSC^hi^;CD166^hi^ generated spheres and LSC^hi^;CD166^lo^ generated spheres (Figure S1B and S1C). Similar to the LSC^hi^ subpopulation [26], castration also leads to significant enhancement of the LSC^hi^;CD166^hi^ sub-population (Figure 1C).

### CD166^hi^ Human Prostate Cells Have Higher Sphere Forming and Regeneration Potential

**Figure 2:**
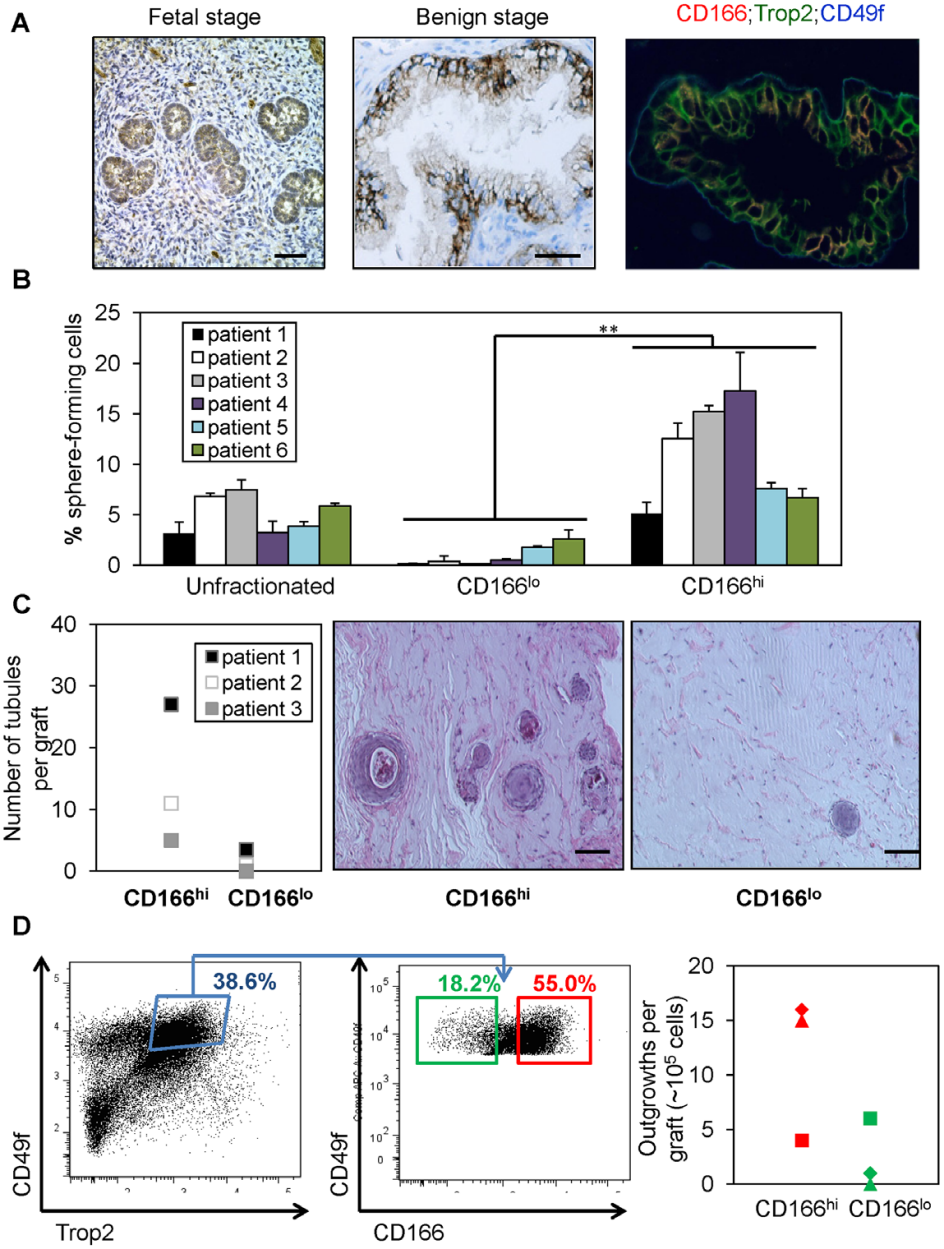
CD166^hi^ human prostate cells have higher sphere forming capacity *in vitro* and more graft outgrowth *in vivo*. (A) IHC staining of CD166 on human fetal prostate tissue and patient prostate cancer tissues. Scale bar: 50 µm. (B) Total dissociated prostate cells, CD166^hi^ and CD166^lo^ populations were isolated by FACS from 6 patient samples. Graph shows the percentage of sphere-forming cells, based on the spheres from each population per 5,000 cells plated after 7 days of culture. Data shown as mean +/− STD (**, p<0.001). (C) CD166^hi^, and CD166^lo^ populations were isolated by FACS from 3 patient samples. CD166^hi^ and CD166^lo^ cells (2×10^5^) were implanted subcutaneously into NOD-SCID/IL2rγ null mice, in combination with 2×10^5^ rUGSM inductive mesenchymal cells. Grafts were harvested, fixed and analyzed after 8–16 weeks. Left, graph shows that CD166^hi^ human prostate cells can form more tubules in graft regeneration assay compared to CD166^lo^ human prostate cells. Right, H&E staining of representive graft. Scale bar: 100 µm. (D) Left, FACS plots show gates drawn for sorting of LTC (TROP2^hi^;CD49f^hi^) CD166^hi^ and LTC;CD166^lo^ subpopulations from one patient. Right, representative graph shows that LTC;CD166^hi^ human prostate cells can form more tubules in graft regeneration assay compared to LTC;CD166^lo^ human prostate cells.

Certain cell surface markers, such as Sca-1, are only expressed in the mouse and therefore cannot be used for isolation of human stem/progenitor cells. CD166, on the other hand, is expressed in various human organs and upregulated in human cancers, including prostate cancer [27]. To determine whether CD166 can be used for enriching human prostate stem/progenitors, we first examined its expression and found that CD166 is highly expressed in the developing human fetal prostate epithelium (Figure 2A, left panel) and focally expressed in the benign adult prostate, which overlaps with a subset of TROP2 and CD49f – positive cells (Figure 2, middle and right panels).

We then evaluated whether CD166 could be used as a marker for enriching human stem/progenitor cells. Benign regions of prostate tissue were collected from multiple patients who underwent radical prostatectomy and dissociated to single cells. Consistent with our previous studies [13], [28], the percentages of CD166^+^ cells vary from patient to patient (data not shown). However, the majority of sphere forming activity was identified in the CD166^hi^ population (Figure 2B), similar to our findings with murine prostate cells. Data are shown from 6 representative patients.

To evaluate whether CD166 can enrich human prostate tissue regeneration capacity *in* *vivo*, benign human prostate cells were dissociated and sorted according to cell surface CD166 expression levels. Equal number of viable CD166^hi^ and CD166^lo^ cells (2×10^5^) was implanted subcutaneously into NOD-SCID/IL2rγ null mice, in combination with 2×10^5^ rUGSM inductive mesenchymal cells. After 8–16 weeks, grafts were harvested, fixed and embedded in paraffin for quantification and analyses. CD166^hi^ cells have more tissue regeneration capacity as evidenced by increased number of tubule-like epithelial structures found in the grafts, which is rarely seen in the CD166^lo^ grafts (Figure 2C). Further analyses showed that the tubule-like structures initiated by CD166^hi^ cells contain CK5 and p63 expressing basal cells, CK8 luminal cells and AR positive cells (Figure S2).

Combination of markers TROP2 and CD49f can separate lineage-negative human prostate epithelial cells into various subpopulations, with TROP2^hi^;CD49f^hi^ (Lin^-^T^hi^C^hi^ or LTC) cells possessing the highest sphere forming capability *in vitro* [29]. Additionally, LTC cells can develop cancer-like phenotype *in vivo* following oncogenic transformation [13]. We tested whether CD166 can further segregate this LTC population. FACS analysis of benign human prostate cells indicated that more than 50% of LTC stem/progenitor cells also express the CD166 surface marker (Figure 2D, left and middle panel). Furthermore, we examined if differences in regeneration potential exist between these two subpopulations. Sorted LTC;CD166^hi^ and LTC;CD166^lo^ cells were injected subcutaneously into NOD-SCID/IL2rγ^-^ null mice with 2×10^5^ rUGSM cells and analyzed 8–16 weeks later. Our *in vivo* data suggest that LTC;CD166^hi^ cells can induce more tubule-like structures, whereas LTC;CD166^lo^ cells have less regeneration capacity (Figure 2D, right panel).

### CD166 can be used to Enrich Tumor Sphere-forming Cells in the *Pten* Null Prostate Cancer Model

**Figure 3:**
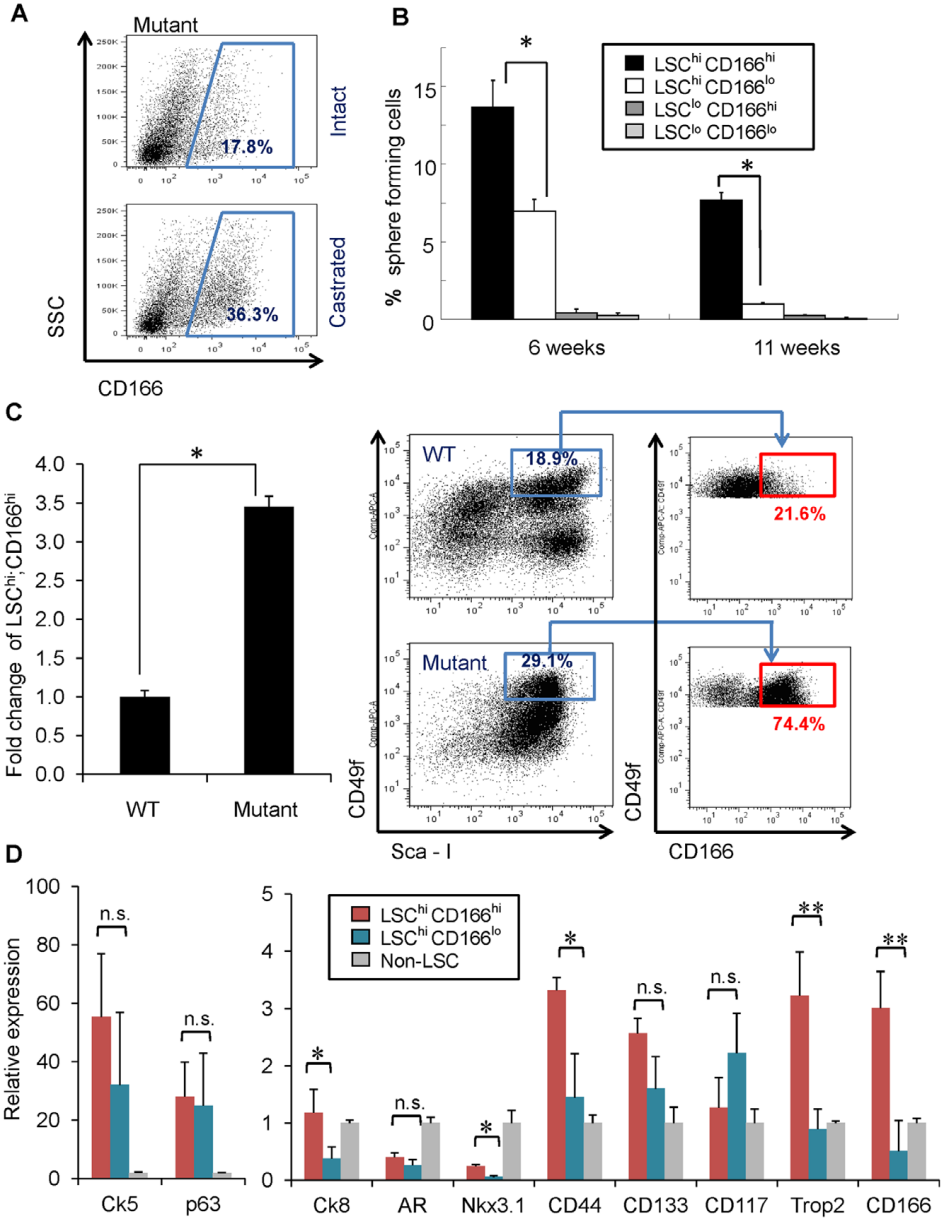
CD166 can be used to enrich tumor initiating cells in *Pten* mutant prostate. (A) FACS blots show increased Lin^-^CD166^hi^ population after castration of *Pten* mutant mice compared to intact *Pten* mutant mice. (B) Four subpopulations (LSC^hi^CD166^hi^, LSC^hi^CD166^lo^, LSC^lo^CD166^hi^, LSC^lo^CD166^lo^) were isolated from *Pten* mutant prostate from either 6 weeks or 11 weeks old mice. Graph shows the percentage of sphere-forming cells. Data from several experiments were pooled. Data shown as mean +/− STD (*, p<0.05, n = 3). (C) Left: bar graph shows fold change of *Pten* mutant LSC^hi^CD166^hi^ content compared to WT; right, FACS blots show the expansion of LSC^hi^ CD166^hi^ cells within LSC population on *Pten* mutant compared to WT. (D) RNA was isolated from non-LSC, LSC^hi^CD166^hi^, and LSC^hi^CD166^lo^ fractions in duplicate experiments. RNA was synthesized into cDNA and subjected to qRT-PCR. Graph shows fold-enrichment over the non-LSC cells for each gene. Gadph was used as the reference gene (*, p<0.05; **, p<0.01; n.s., not significant).

To examine whether CD166 can enrich tumor initiating cells after castration, we compared the percentage of CD166^hi^ subpopulation between intact and castrated *Pten* mutant mice and observed the expansion of CD166^hi^ subpopulation after castration (Figure 3A). Next, we compared the sphere formation capabilities of LSC^hi^;CD166^hi^, LSC^hi^;CD166^lo^, LSC^lo^;CD166^hi^, and LSC^lo^;CD166^lo^ subpopulations at the pre-cancer PIN (6 weeks) and cancer stages (11 weeks). We found that the LSC^hi^;CD166^hi^ subpopulation has much higher sphere-forming ability, and nearly all sphere-forming activity in the cancer stage resides in the LSC^hi^;CD166^hi^ subpopulation (Figure 3B). Consistent with our previous observation that *Pten* mutant spheres are larger than WT control spheres [19], both LSC^hi^;CD166^hi^ and LSC^hi^;CD166^lo^ subpopulations form large prostate spheres (Figure S3). Our previous study suggested that *Pten* deletion promotes the expansion of LSC^hi^ prostate stem/progenitor cells [18], [19]. Within the LSC^hi^ population, we observed selective expansion of LSC^hi^;CD166^hi^ cells. *Pten* mutant mice have more than a 3-fold increase in the percentage of LSC^hi^;CD166^hi^ subpopulation, compared to WT littermates (Figure 3C).

To further study the LSC^hi^;CD166^hi^ subpopulation, we isolated RNA from LSC^hi^;CD166^hi^, LSC^hi^;CD166^lo^ subpopulations and the cell fraction depleted of LSC cells (non-LSC^hi^) and compared their gene expressions by RT-PCR analysis. LSC^hi^;CD166^hi^ subpopulation expresses similar levels of basal cell markers *Ck5* and *p63* as the LSC^hi^;CD166^lo^ subpopulation (Figure 3D, left panel). However, LSC^hi^;CD166^hi^ subpopulation expresses much higher level of luminal marker *Ck8* and *Trop2*, a new epithelial surface marker we recently identified for enriching stem cell activities in both murine and human prostates [13], [29] (Figure 3D, right panel). Further examination of several other epithelial cell stem cells markers [10], [30], [31], [32], [33] showed that LSC^hi^;CD166^hi^ cells have significantly higher *CD44* and *Nkx3.1* expression compared to LSC^hi^;CD166^lo^ cells. Although compared to non-LSC population, LSC^hi^;CD166^hi^ cells express less *Nkx3.1*. No significant differences were found in *CD117*, and *CD133* expressions between these two populations (Figure 3D, right panel).

### CD166 Expression is Upregulated in Human Castration Resistant Prostate Cancer

**Figure 4:**
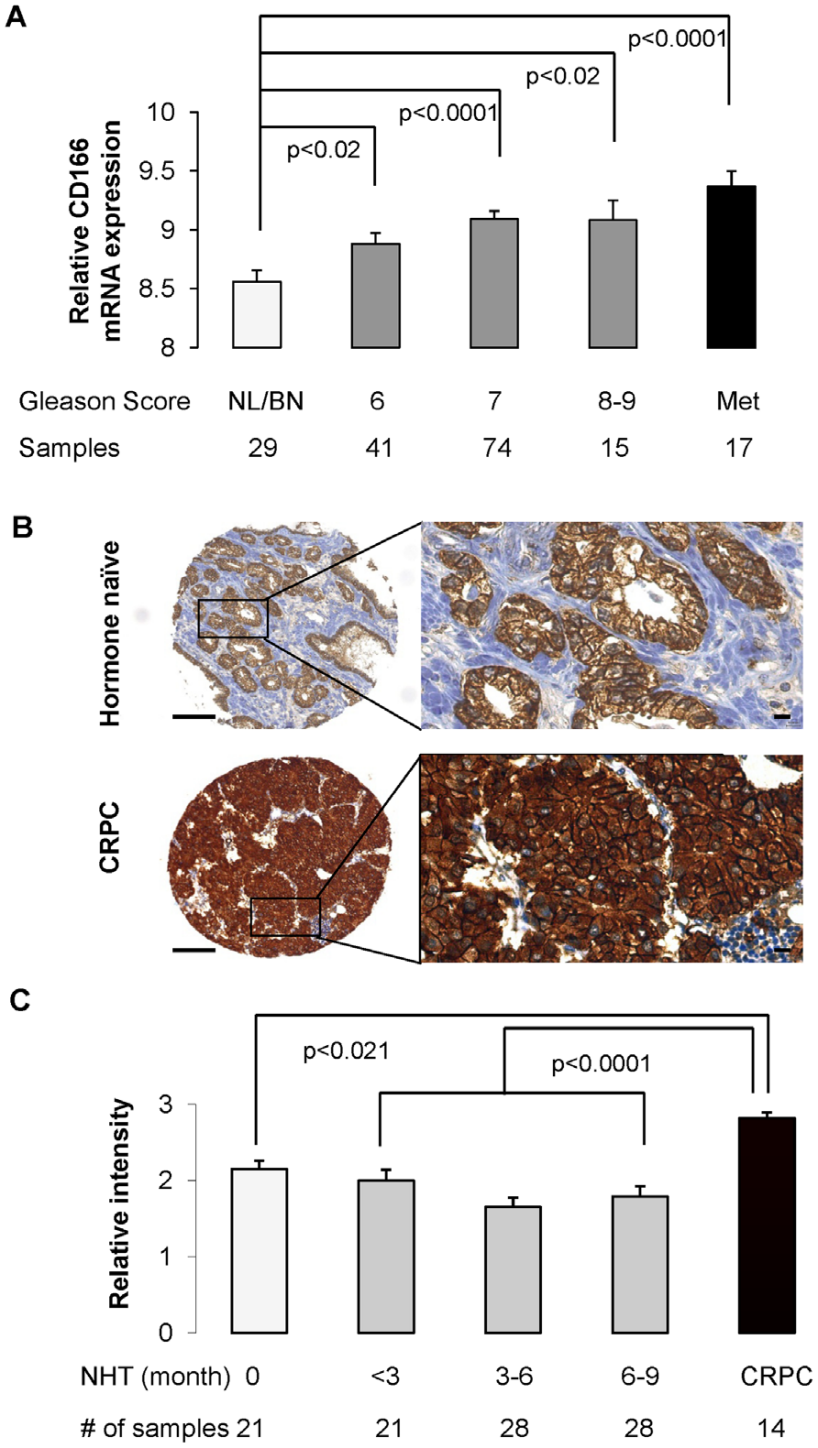
Gene expression profiling and tissue microarray (TMA) demonstrates that increased CD166 expression is correlated with high Gleason score and human castration resistant prostate cancer. (A) CD166 gene expression from 147 human prostate tumors was analyzed by comparing different Gleason score groups to normal/benign (NL/BN) prostate. (B) Representative IHC staining of CD166 expression from human prostate TMA. Top: hormone naïve primary prostate cancer; Low: castration resistant prostate cancer showing highly intensive immunostaining. Scale bar: 100 µm (left); 10 µm (right). (C) Data from 112 samples were calculated and statistical analysis of CD166 expression of human TMA conducted. NHT: neoadjuvant hormone therapy; CRPC: castrate resistant prostate cancer. Column, mean CD166 staining in NHT and CR tissues. Samples were graded from 0 to +3 representing the range from no staining to heavy staining by visual scoring. Error bar: standard error. Immunoreactivity of CD166 is significantly higher in CRPC group compared with untreated group (p<0.021) or NHT with different treatment times (p<0.0001).

Having found that CD166 can be used to enrich for human LTC cells and mouse tumor in itiating cells, we then examined the relationship between CD166 expression and human prostate cancer progression. In clinically annotated data of 218 prostate tumors [34], CD166 gene expression significantly correlates with increased prostate cancer aggressiveness, as indicated by Gleason score, with highest expression in metastasis samples (Figure 4A). We further surveyed CD166 expression on human prostate cancer tissue microarrays, which consist of 14 castration resistant (CRPC) metastasis samples and 98 hormone naïve primary cancer samples from patients receiving either neoadjuvant hormone treatment (NHT) for various periods or receiving no treatment. CD166 is significantly enhanced in CRPC samples (Figure 4B for representative images). Compared to the predominant membrane localization of CD166 in hormone naïve primary cancer samples, we observed intense cytoplasmic localization of CD166 in CRPC bone metastasis samples (Figure 4B, high magnification). CD166 expression levels were scored and p values are computed by Mann-Whitney test. CD166 protein expression level is significantly higher in CRPC samples as compared with primary cancers with (p<0.0001) or without (p<0.02) NHT (Figure 4C). These data suggest that CD166 is a castration-enriched marker for both murine and human prostate cancer.

### Loss of CD166 does not Interfere with WT Prostate Development and Prostate Sphere Formation

**Figure 5:**
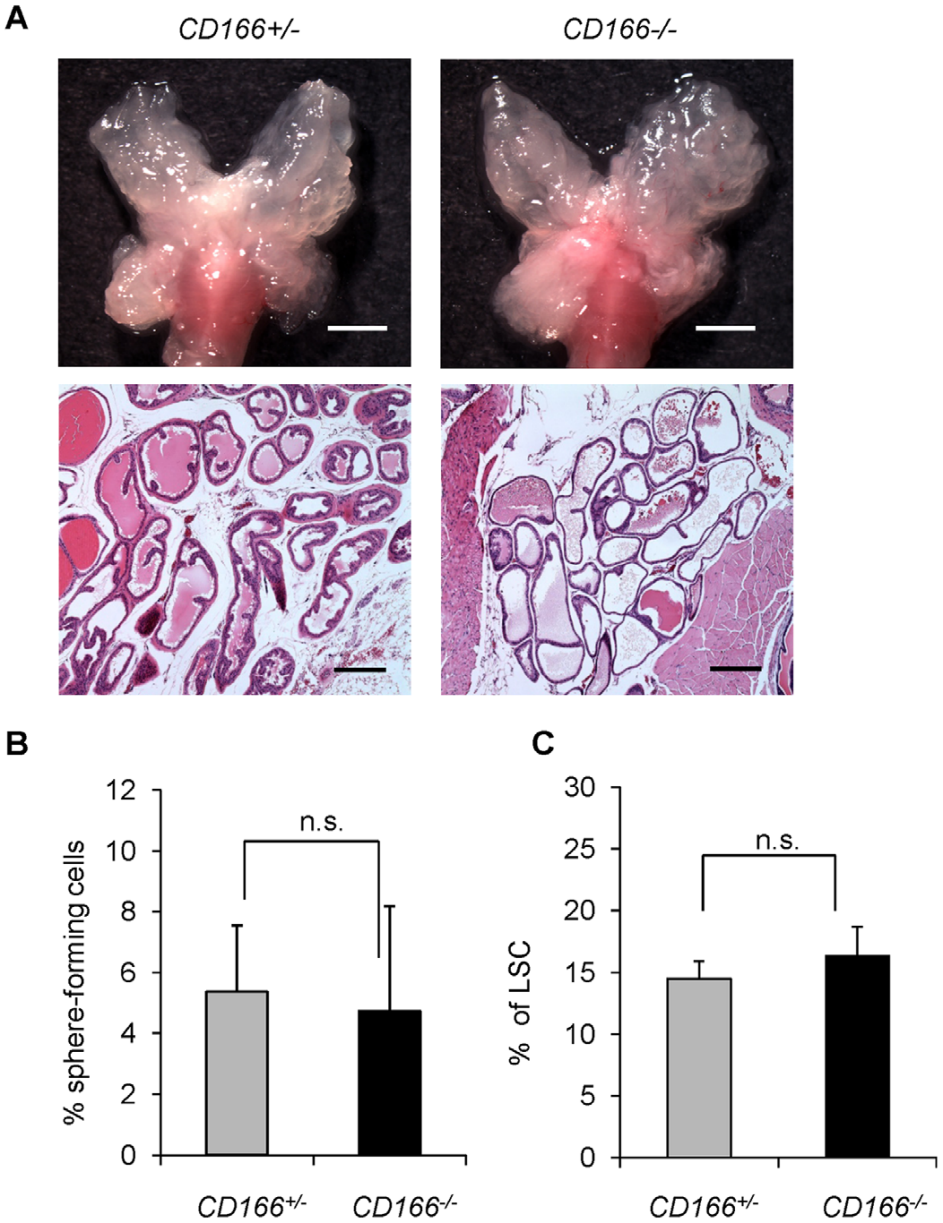
Loss of CD166 does not block WT prostate development and stem/progenitor cell function. (A) Top: The gross anatomy of the prostate of *WT* and *CD166−/−* mice at 8 weeks of age, scale bar: 2 mm. Bottom: HE staining of DLP section from WT and *CD166* ^−/−^ mice at 8 weeks of age, scale bar: 200 µm. (B) Comparison of sphere formation from total unsorted prostate cells (5000 per 12-well) between *CD166^+/−^* and *CD166^−/−^* prostates. Data represented as mean +/− STD (p>0.05, n = 3). (C) Comparison of LSC^hi^ content between *CD166^+/−^* and *CD166^−/−^* prostates at 8–12 weeks age (p>0.05, n = 5).

While expressed in a wide variety of tissues, CD166 is usually restricted to subsets of cells involved in dynamic growth and/or migration, including neural development, branching organ development, hematopoiesis and immune response [27]. To test whether CD166 plays an intrinsic role in regulating prostate stem/progenitor cells, we analyzed *CD166* knockout mice (*CD166^−/−^*). Genetic deletion of *CD166* gene was achieved by replacing its first exon with a cDNA encoding EGFP [35]. *CD166* null mice are phenotypically normal and fertile [35]. We examined the prostate at 8 and 20 weeks of age and found no difference in gross anatomy and histology among WT (data not shown), *CD166^+/−^* and *CD166^−/−^* mouse prostates (Figure 5A).

To further examine whether loss of CD166 has any effect on prostate stem/progenitor cells, we compared sphere formation activities of *CD166^+/−^* and *CD166^−/−^* prostate epithelium and found there is no significant difference (Figure 5B). In addition, spheres generated from *CD166^−/−^* prostate have similar size distribution compare to those from *CD166^+/−^* prostate epithelium (data not shown). Similarly, FACS analysis demonstrated that loss of CD166 does not affect LSC^hi^ content of prostates isolated from the *CD166^−/−^* mice (Figure 5C), suggesting that CD166 does not play an essential role in normal prostate gland development or prostate stem/progenitor number and function.

### Genetic Deletion of *CD166* does not Block Prostate Cancer Progression

**Figure 6:**
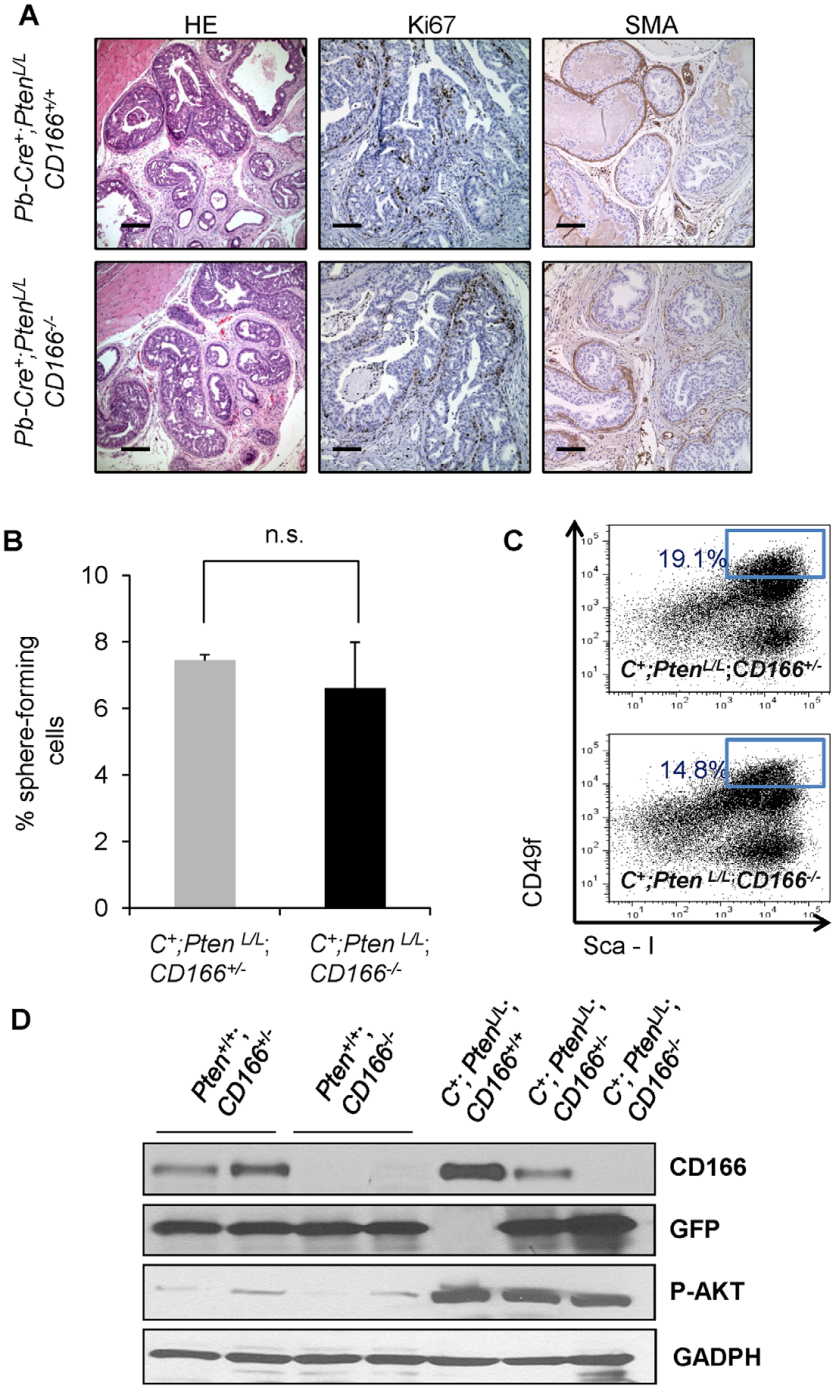
Loss of CD166 does not block prostate tumor progression and tumor initiating cell function in *Pb-Cre^+^;Pten^L/L^;CD166* ^−/−^ mice. (A) Evaluation of *CD166* deletion on prostate cancer progression (HE staining, scale bar: 200 µm), cell proliferation (Ki67 staining, scale bar: 100 µm), and prostate tumor invasion (SMA staining, scale bar: 100 µm) by comparing age matched *Pb-Cre^+^, Pten^L/L^, CD166^+/+^* and *Pb-Cre^+^, Pten^L/L^, CD166* ^−/−^ prostate tissue at 20 weeks of age. (B) Comparison of sphere formation from total unsorted prostate cells (5000 per 12-well) between *Pb-Cre^+^, Pten^L/L^, CD166^+/−^* and *Pb-Cre^+^, Pten^L/L^, CD166^−/−^* prostate (9 weeks of age). (C) A representative FACS blot shows LSC content between *Pb-Cre+, Pten^L/L^, CD166^+/−^* and *Pb-Cre^+^, Pten^L/L^, CD166^−/−^*. (D) Examination of protein levels of CD166, P-AKT and GFP among different prostate tissue with indicated genotype by Western blotting. GADPH is included as an equal loading control.

It has been postulated that CD166 functions as a cell surface sensor for cell density and controls the transition between local cell proliferation and tissue invasion during melanoma progression [36]. To examine whether CD166 plays an essential role in prostate cancer development, especially in the tumor initiating cells, we crossed *CD166^−/−^* mice with the *Pten* conditional knockout mice [17]. Histopathologic analysis indicated that loss of CD166 did not significantly change the kinetics of prostate cancer development in *Pten* null model and all *Pb-Cre^+^;Pten^L/L^;CD166^−/−^* mice developed adenocarcinoma around 9 weeks of age (Figure 6A and data not shown). We observed similar levels of Ki67^+^ cells between *Pb-Cre^+^,Pten^L/L^,CD166^+/+^ and Pb-Cre^+^;Pten^L/L^; CD166^−/−^* prostates (Figure 6A). SMA staining also demonstrated that loss of CD166 does not block prostate cancer cells from local invasion (Figure 6A, right panels).

We then compared the sphere formation between *Pb-Cre^+^;Pten^L/L^; CD166^+/−^* and *Pb-Cre^+^;Pten^L/L^;CD166^−/−^* prostates and found that loss of CD166 does not interfere with sphere-forming activity of *Pten* null epithelium (Figure 6B). Moreover, *CD166^−/−^* prostates have similar LSC^hi^ content as compared to *CD166^+/−^ Pten* null prostates (Figure 6C). Since PI3K/AKT pathway activation is a driving force for cell proliferation and prostate cancer progression in *Pb-Cre^+^;Pten^L/L^* prostate cancer [17], [20], we then examined whether there is any alteration of AKT activation after genetic deletion of *CD166*. Western blot analysis demonstrated that *Pb-Cre^+^;Pten^L/L^;CD166^−/−^* prostate has no CD166 expression, but has similar P-AKT levels compared to *Pb-Cre^+^;Pten^L/L^;CD166^+/+^* and *Pb-Cre^+^;Pten^L/L^;CD166^+/−^* prostate (Figure 6D). We further confirmed that there is no negative selection against *Pten^−/−^;CD166^−/−^* cells since equal intensity of knockin-GFP protein can be detected in all cohorts except *CD166^+/+^* mice.

**Figure 7:**
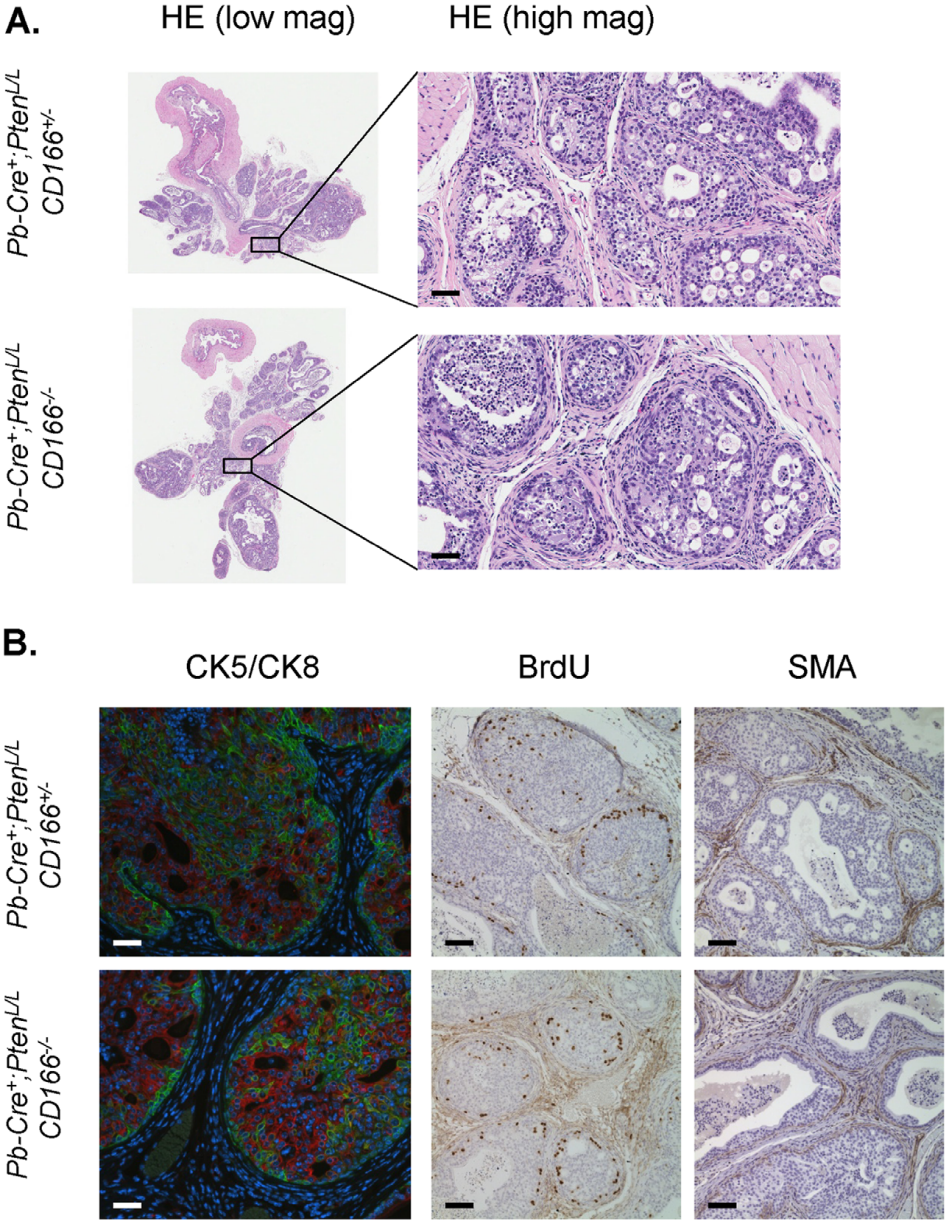
Loss of CD166 does not block castration resistant prostate tumor progression in *Pb-Cre^+^;Pten^L/L^;CD166* ^−/−^ mice. *Pb-Cre^+^, Pten^L/L^, CD166^+/−^* and *Pb-Cre^+^, Pten^L/L^, CD166* ^−/−^ mice were castrated at the age of 12 weeks using standard techniques. At 8 weeks post-castration, mice were intraperitoneal injected with a single dose of 100 µl (1 mg) of BrdU solution and sacrificed 4 hour later for analysis. Evaluation of the effects of *CD166* deletion on (A) castration resistant prostate cancer progression (HE), and (B) cell lineage composition (CK5/CK8), cell proliferation (BrdU) and prostate tumor invasion (SMA) were performed. Scale bar: 50 µm.

Since we see significant overexpression of CD166 in human CRPC samples, we next investigated whether CD166 would influence the development of CRPC in the *Pten* null prostate cancer model. *Pb-Cre^+^;Pten^L/L^;CD166^+/−^* and *Pb-Cre^+^;Pten^L/L^;CD166^−/−^* males were castrated at 12 weeks and prostates were isolated 8 weeks later. As shown in Figure 7, deletion of CD166 does not significantly influence the formation of CRPC, as evidenced by similar pathohistology (Figure 7A), CK5/CK8 marker distribution, BrdU pulse labeling and SMA staining in both cohorts (Figure 7B). Taken together, our genetic studies indicate that CD166 has limited intrinsic function in the prostate, even in the tumor initiating cells.

## Discussion

Few surface markers are currently available for enriching both murine and human prostate tissue stem/progenitor cells and for identifying prostate cancer initiating cells. By searching for those cell surface molecules that are upregulated in castrated murine prostate and castration resistant prostate cancers (CRPC) of murine and human origins, we identified CD166 as a surface marker for enriching both murine and human prostate tissue stem/progenitor cells based on *in vitro* sphere forming and *in vivo* tissue regeneration analyses. Importantly, upregulated CD166 expression and expansion of CD166^hi^ cells correlate with *Pten* null CRPC progression as well as human CRPC development, although genetic deletion of *CD166* does not interfere with normal murine prostate development or *Pten* null prostate cancer progression. Together, our study suggests CD166 can be used as a potential surface marker for identifying castration resistant tumor cells for targeted drug delivery.

CD166 expression has been proposed as a prognostic marker for several cancers, including breast [37], prostate [38], ovarian [39], pancreatic [40], colon [41], oral cancers [42], melanoma [36] and gastric cancers [43]. Importantly, our microarray and TMA studies demonstrate the association of increased CD166 expression with human prostate cancer metastasis and CRPC development. Moreover, within both murine and human prostates, we show that the CD166-high expressing subpopulation encompasses prostate stem/progenitor and cancer initiating cells.

To investigate human prostate tissue stem/progenitor cell properties, we evaluated adult human prostate epithelium dissociated from benign prostate, rather than cell lines and xenografts. The advantage of this approach is to maintain the original heterogeneity in human prostate samples by avoiding the effect of long-term *in vitro* selection. However, there appears to be greater variability among patient samples in the tissue regeneration assays. This may be due to the difference in sample variability (i.e., ischemia time prior to tissue processing and cell retrieval), individual variability in CD166 expression, and technical challenges related to the tissue regeneration assays using human prostate cells. Therefore, analysis of sufficient patient samples is essential in order to draw a valid conclusion. In the current study, 6 human samples were utilized for the *in vitro* sphere forming and another 6 samples were used for *in vivo* regeneration assays. Using this system, we have previously defined TROP2^hi^;CD49f^hi^ as a cancer initiating cell (cell of origin) for human prostate cancer [13]. In the current study, CD166^hi^ population demonstrated significantly increased sphere-forming capacity compared to the patient-matched CD166^lo^ population. In addition, our study demonstrates that CD166 can not only enrich human sphere-forming cells, but also segregate TROP2^hi^;CD49f^hi^ into two functionally different populations, with TROP2^hi^;CD49f^hi;^CD166^hi^ having higher regeneration capacity *in vivo*, compared to TROP2^hi^CD49f^hi^CD166^lo^. CD166 is also highly upregulated in CRPC based on our gene expression analysis and tissue microarray study. Therefore, CD166 may enrich both human prostate tissue stem/progenitor cells and castration resistant prostate cancer cells.

LSC^hi^ subpopulation has been defined as the murine prostate tissue stem/progenitor cells and expands significantly following castration [12], [19], [26]. LSC^hi^ cells express basal markers and demonstrated robust sphere-forming activity *in vitro* and prostate regeneration capability *in vivo* [26]. In contrast to luminal cells, LSC^hi^ cells respond efficiently to multiple oncogenic insults for prostate cancer initiation using a transplantation-based prostate regeneration assay [12]. We and others have demonstrated that the LSC^hi^ population, isolated from *Pten* null prostate tissue, is sufficient to regenerate cancerous morphology upon transplantation that closely mimics that of primary cancers [19], [44]. In this study, we further separated LSC^hi^ subpopulation into CD166^hi^ and CD166^lo^ subsets and found that most of sphere-forming activities are associated with the LSC^hi^;CD166^hi^ cells. Importantly, this LSC^hi^;CD166^hi^ population was demonstrated to have self-renewal activity as spheres from this population could be passaged at least 3 generations with a high rate of sphere formation. Moreover, LSC^hi^;CD166^hi^ cells are expanded upon castration as well as *Pten* deletion in comparison to LSC^hi^;CD166^lo^ cells. Therefore, CD166 can further enrich murine prostate cancer initiating cells and castration resistant cells.

The relationship of LSC^hi^;CD166^hi^ cancer initiating cells described here to other cell populations is of obvious interest [45]. Using lineage tracing and cell type-specific Cre lines, a recent report demonstrates that both luminal cells and basal cells can initiate prostate cancer upon *Pten* deletion [14]. This new observation is not in conflict with our previous studies: we showed that *Pten* deletion mediated by *Pb-Cre* happens in both basal and luminal cells [18]. In addition, we observed significant expansion of a subset of prostate cancer cells positive for basal cell markers CK5 and p63 and luminal cell marker CK8, suggestive of transient amplifying/intermediate cells [18], [46]. Compared to LSC^hi^;CD166^lo^ cells, one of the distinguishing features of LSC^hi^;CD166^hi^ cells is the higher *Trop2* expression, a cell surface marker we have used for enriching both murine and human tissue stem cells [13], [29]. TROP2 can functionally segregate mouse LSC population but there is no cytokeratin phenotypic difference between LSC^hi^;Trop2^hi^ and LSC^hi^;Trop2^lo^ population [29]. CD166, on the other hand, can enrich *Pten* null LSC^hi^ population with CK5^+^/p63^+^/CK8^+^/AR^−/^TROP2^hi^ characteristics, suggesting that CD166 may preferentially enrich for CK5^+^/CK8^+^ transient amplifying/intermediate cells, which currently cannot be prospectively purified. Increased CK5^+^;CK8^+^ cells have been observed in the *Pten* conditional knockout model [18], [47] as well as *Pten^−/−^;TP53^−/−^* prostates cancer model [48]. A recent study also identified a subset of tumor-initiating stem-like cells in human prostate cancer cell lines and xenografts based on co-expression of the human pluripotent stem cell marker TRA-1-60, CD151 and CD166 [49]. Interestingly, this subtype of human prostate tumor initiating cells also have the AR^−^;CK5^+^;CK8^+^ phenotype [49]. Another characteristic of LSC^hi^;CD166^hi^ cells is relatively higher CD44 expression. Since knockdown of CD44 was very effective to suppress cancer stem cell regeneration and metastasis [30], it will be interesting to examine whether there is any functional role for CD44 in LSC^hi^;CD166^hi^ tumor initiating cells.

As an adhesion molecule, CD166 can initiate homophilic (CD166-CD166) or heterophilic interaction (CD166-CD6), and play important roles in neural guidance and the immune system [27]. CD166 has also been suggested to play a critical role in various human cancers and as a potential therapeutic target for cancer initiating cells, similar to CD44 [30] and CD47 [50]. A truncated CD166 variant has been shown to block melanoma metastasis by interfering with the CD166-CD166 homophilic interaction [51]. Similarly, novel human recombinant single-chain anti-CD166 antibodies have been shown to inhibit colorectal carcinoma growth as well as breast cancer cell invasion [52]. Unlike subcutaneous allograft or xenograft models used in above studies, we defined the functions of CD166 in prostate cancer initiating cells and prostate cancer development in immune competent mice within the natural prostate environment. By generating the *Pb-Cre^+^;Pten^L/L^*;*CD166^−/−^* line, our study demonstrates that loss of CD166 within LSC^hi^ population does not change their ability to form spheres *in vitro* and block prostate cancer initiation and progression *in vivo*. As it is possible that other members of the Cell Adhesion Molecule (CAM) family can compensate for the role of CD166 in murine prostate cancer development, we cannot conclude that CD166 has no *in vivo* function on prostate cancer initiation. Nevertheless, since cancer initiating cell surface markers can be used for molecular imaging [53] and/or for internalizing a death-inducing compound for targeted therapies [54], our work suggests that CD166 may be for a suitable surface marker for future targeted drug delivery [55]. Recently, a promising study showed substantial cytotoxic effects of the CD166 scFv-condugated drugs on three human prostate cancer cell lines (Du-145, PC3, and LNCaP) [55]. Since CD166 is highly expressed on both human and mouse tissue stem/progenitor cells, it will be interesting to examine the effect of this targeted drug delivery on their prostate sphere forming activity and prostate regeneration potential. The *Pb-Cre^+^;Pten^L/L^*;*CD166^+/−^* and *Pb-Cre^+^;Pten^L/L^*;*CD166^−/−^* mouse models generated in this study, therefore, can be used to investigate the efficiency of CD166 - mediated drug delivery to prostate cancer initiating cells *in vivo*, especially during CRPC development.

## Materials and Methods

### Mouse Strains

Mutant mice with prostate-specific deletion of *Pten* were generated as described previously under a mixed background [17]. The 129/C57 background CD166 knockout (*CD166^−/−^*) was generously provided by the laboratory of Dr. Weiner of University of Iowa [35]. *Pten^L/L^* mice on a 129/Balb/c background were first crossed to the *CD166^−/−^* mice [35] to get F2 female *Pten^L/L^;CD166* ^−/−^. *Pb-Cre* ^+^; *Pten^L/L^*;*CD166* ^−/−^ mice were then generated by crossing female Cre^-^ *;Pten^L/L^*;*CD166* ^−/−^ with male *Pb-Cre* ^+^;*Pten^L/L^*;*CD166* ^+/−^. All animal experiments were performed following Institutional Approval for Appropriate Care and use of Laboratory animals by the UCLA Institutional Animal Care and Use Committee (Chancellor's Animal Research Committee (ARC)), Animal Welfare assurance number A3196-01.

### Tissue Collection and FACS

The preparation of prostate epithelial cell suspensions from male mice were described previously [20]. Dissociated prostate cells were suspended in DMEM/10% FBS and stained with antibody for 15 min at 4°C. Antibodies are listed in Table S2. FACS analysis was performed by using BD FACS Canto (BD Biosciences, San Jose, CA). Cell sorting was done by using BD FACS Vantage and the BD FACS Aria II.

### *In vitro* Prostate Sphere-forming Assays

Prostate spheres were cultured and passaged as described previously [56], [57]. FACS-isolated prostate cells or unsorted prostate cells were counted and suspended into a 100 µL mixture of 1∶1 Matrigel (BD Biosciences, San Jose, CA) and PrEGM (Lonza, Walkersville, MD). Samples were plated around the rims of wells in a 12-well plate and allowed to solidify at 37°C for 45 minutes, before 1 ml of PrEGM was added. Sphere media was changed every three days. Spheres were counted after 8 days. To passage spheres, medium was aspirated off and matrigel was digested with 1 mL Dispase solution (Invitrogen, Carlsbad, CA, 1 mg/ml, dissolved in PrEGM medium) for 30 minutes at 37°C. Spheres were collected, incubated in 1 ml warm Trypsin/0.05% EDTA at 37°C for 5 minutes, passed through a 27-gauge syringe 5 times, and filtered through a 40 µm filter. Cells were counted by hemocytometer and replated.

### RNA Isolation and qRT-PCR

Sorted cells were collected and spun down. RNAs from sorted cells were extracted using TRIzol® Reagent (Invitrogen, Carlsbad, CA). RNAs were reverse transcribed into cDNA with SuperScript III First-Strand Synthesis System for qRT-PCR (Invitrogen, Carlsbad, CA), and quantitative PCR was done in the iQ thermal cycler (Bio-Rad) using the iQSYBR Green Supermix (Bio-Rad) in triplicate. Primers used for study are Ck5 (F5′-ACCTTCGAAACACCAAGCAC-3′; R5′-TTGGCACACTGCTTCTTGAC-3′), Ck8 (F5′-ATCGAGATCACCACCTACCG-3′; R5′-TGAAGCCAGGGCTAGTGAGT-3′), p63 (F5′-CCCACAGACTGCAGCATTG-3′; R 5′-GAGATGAGGAGGTGAGGAGAAG-3′), AR (F5′-AACCAACCAGATTCCTTTGC-3′; R5′-ATTAGTGAAGGACCGCCAAC-3′), CD166 (F 5′-CCTAAGAGAGGAGCGGATTG-3′; R5′-CAGCCACTCCCAGAACAAAG-3′), Trop2 (F5′- AGACCAAAGCCTGCGCTGCG-3′; R 5′- AGCTGGGGTGCAGCTTGTAG-3′), Gadph (F5′-ACTGGCATGGCCTTCCG-3′; R5′-CAGGCGGCACGTCAGATC-3′), CD117 (F5- AGAAGCAGATCTCGGACAGC-3′; R5′- GACTTGGGTTTCTGCTCAGG-3′), CD133 (F5-ACCAACACCAAGAACAAGGC-3′; R5′-GGAGCTGACTTGAATTGAGG-3′), CD44 (F5- GTCAACCGTGATGGTACTCG-3′; R5′-AGTGCACAGTTGAGGCAATG-3′), Nkx3.1 (F5’-TCCGTCTTTTGGCTCTGAGT-3′; R5′- GTGAAAGTGCACGCTGAAAA-3′).

### Immunofluorescence and Immunohistochemistry Analyses

Tissue analysis was carried out using standard techniques as described previously [17]. Sections (4 µm) were stained with hematoxylin and eosin (H&E) or with specified antibodies (Table S2).

### Western Blot Analysis

Total protein was extracted with RIPA buffer (20 mM Tris-HCl, pH 7.5, 150 mM NaCl, 1 mM Na_2_EDTA, 1 mM EGTA, 1% NP-40, 1% sodium deoxycholate) with fresh added phosphatase inhibitors (Sigma, St. Louis, MO) and protease inhibitors (Roche, Indianapolis, IN). Protein concentrations were determined by Bradford Assay kit (BioRad, Hercules, CA). Protein was separated by 4–15% gradient SDS/PAGE (BioRad, Hercules, CA) and transferred onto a PVDF membrane (Amersham Biosciences, Arlington Heights, IL). The membrane was blocked in 5% skim milk, and subsequently incubated with primary antibodies against CD166 and GADPH (Santa Cruz Biotechnology, Santa Cruz, CA), GFP (Abcam, Cambridge, MA), phospho-AKT Ser473 (Cell Signaling Technology, Beverly, MA) at 4°C overnight followed by incubation with peroxidase-conjugated goat anti-mouse IgG or goat anti-rabbit IgG (Jackson ImmunoResearch, Inc., West Grove, PA), and developed with Pierce ECL reagent (Thermal Scientific, Rockford, IL).

### Human Prostate Cancer Tissue Microarray (TMA)

TMA used to survey CD166 expression is composed of 112 patient samples. Written consent was obtained from the patient as well as ethics approval from University of British Columbia-British Columbia Cancer Agency Research Ethics Board (UBC BCCA REB), Vancouver, Canada. The 112 patient specimens were spotted in triplicate to create a tissue microarray with 336 cores as described previously in [58]. Scoring method was based on the intensity of the staining in each core on a 4-point scale from none (0) to high (3). Mann-Whitney test was used to compare CD166 protein expression difference between different groups. p values <0.05 were considered significant.

### Human Prostate Tissue Acquisition and Dissociation

Human prostate tissue was obtained via a research protocol that was approved by the Office for the Protection of Research Subjects at UCLA and the Greater Los Angeles VA Medical Center. Informed written consent was obtained on all participants where identifying information was included. A frozen section was prepared from an adjacent slice of prostate tissue in order to determine the location of tumor nodules. Tumor areas were encircled and dissected away from benign regions within the fresh tissue slice. Benign tissue specimens were placed on ice and brought immediately to the laboratory for mechanical and enzymatic digestion [28]. Prostate tissue was minced into small fragments (1 mm^3^) in RPMI-1640 medium supplemented with 10% FBS and went through through enzymatic digestion (12 h in 0.25% type I collagenase followed by TripLE (Invitrogen) for 5 min at 37°C). Cell suspensions were passed through a 23-gauge needle and were filtered through 40- µm filters. Cells were plated overnight in PrEGM as described above for sphere formation assay or tissue regeneration assay.

### Tissue Regeneration Assay

*In vivo* tissue experiments were performed in male NOD-SCID/IL2rγ null mice in accordance with protocol number 2007-189-11A, approved by the Animal Research Committee within the Office for the Protection of Research Subjects at UCLA. Cells of interest were collected from FACS sorting. 2×10^5^ viable cells were then mixed with 2×10^5^ rat urogenital sinus mesenchyme (rUGSM) and suspended in 100 µL with 50∶50 matrigel:PREGM [4], [28], [59]. Cell/Matrigel mixtures were then injected subcutaneously into NOD-SCID/IL2rγ null mice. Animals were supplemented with a 12.5 mg 90-day release testosterone pellet under the skin (Innovative Research of America, Sarasota, FL). Grafts were harvested 8–16 weeks later and subjected to further analysis.

## Supporting Information

Figure S1 **WT LSC^hi^; CD166^hi^ prostate cells demonstrate higher self-renewal activity.** (A) LSC^hi^;CD166^hi^ and LSC^hi^; CD166^lo^ cells were isolated by FACS from 8- to 10-week-old mice and plated for sphere formation assay. Spheres from the each subpopulation (P0) were dissociated and replated for 3 successive generations (P1–P3). Graph shows the percentage of sphere-forming cells, based on the spheres from each population per 5000 cells plated after 8 days of growth. Error bars represent means and STD from triplicates of one of the two independent experiments (**, *P*<0.001). (B) Comparison of sphere size distribution between LSC^hi^; CD166^hi^ and LSC^hi^; CD166^lo^ formed spheres. n.s., not significant. (C) Representative sphere images of LSC^hi^;CD166^hi^ and LSC^hi^; CD166^lo^ cells generated spheres. Scale bar: 100 µm.
(TIF)

Figure S2 **Immunohistochemical analysis of CD166^hi^ human prostate epithelium-derived graft demonstrates nuclear expression of AR and p63, CK5 and CK8 positive cells and Ki67 positive cells within tubule structure. Scale bar: 50 µm.**
(TIF)

Figure S3 **LSC^hi^;CD166^hi^ and LSC^hi^; CD166^lo^ cells isolated from** ***Pten*** **mutant prostate form spheres with similar size distribution.** Representative sphere images of LSC^hi^;CD166^hi^ and LSC^hi^; CD166^lo^ cells generated spheres. Top: spheres maintained in matrigel. low: spheres released from matrigel after dispase treatment. Scale bar: 200 µm.
(TIF)

Table S1 Compared to intact prostate epithelium WT CD166 gene expression is significantly increased at day 3 post-castration.
(TIF)

Table S2 Antibodies used for FACS, IHC and IF.
(TIF)
