# Supplementary figures and images for: Identification of CD166 as a Surface Marker for Enriching Prostate Stem/Progenitor and Cancer Initiating Cells
Source: PLoS One. 2012 Aug 3;7(8):e42564. doi: 10.1371/journal.pone.0042564 (PMC3411798; doi:10.1371/journal.pone.0042564)

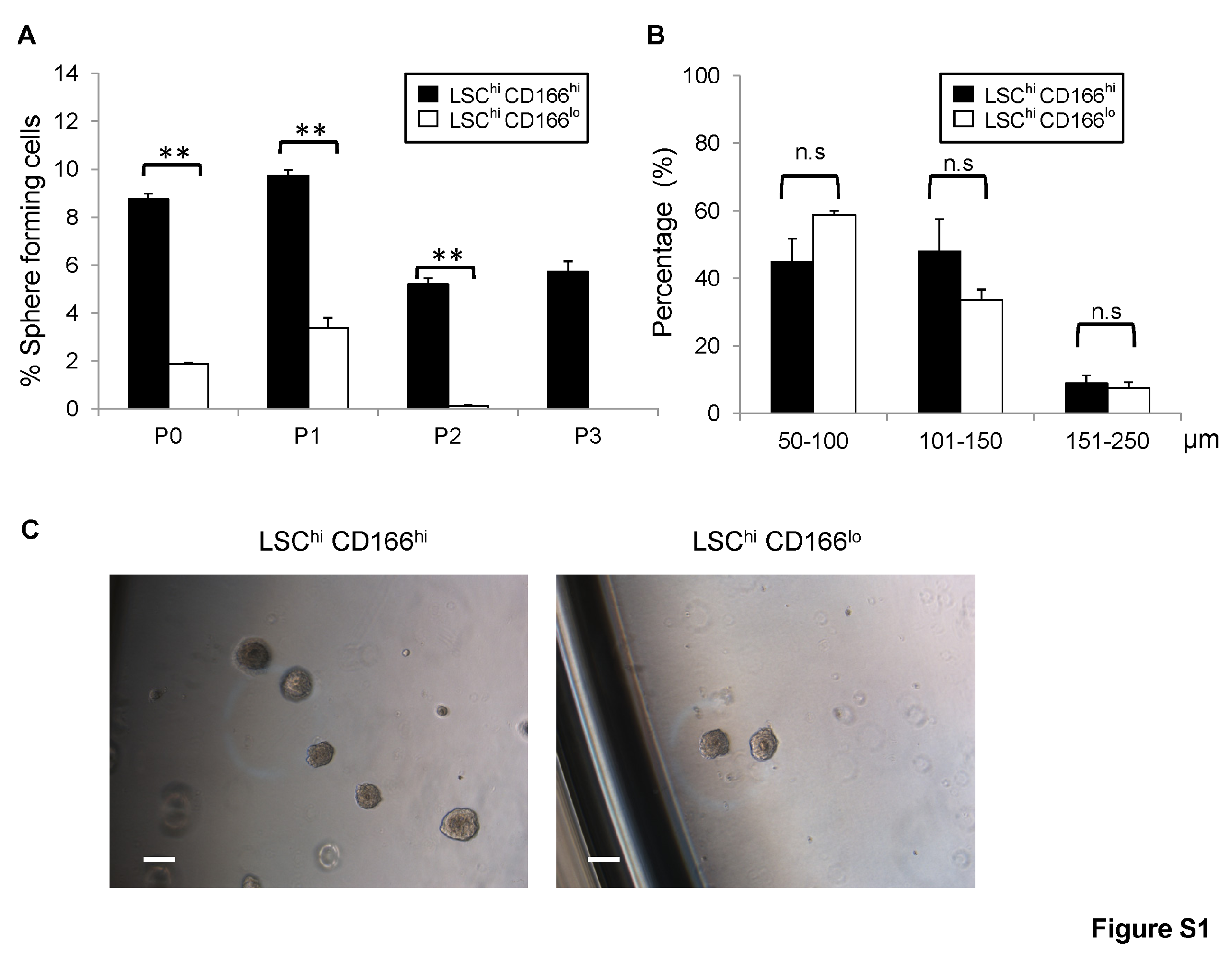

Supplement: Figure S1 — WT LSChi; CD166hi prostate cells demonstrate higher self-renewal activity. (A) LSChi;CD166hi and LSChi; CD166lo cells were isolated by FACS from 8- to 10-week-old mice and plated for sphere formation assay. Spheres from the each subpopulation (P0) were dissociated and replated for 3 successive generations (P1–P3). Graph shows the percentage of sphere-forming cells, based on the spheres from each population per 5000 cells plated after 8 days of growth. Error bars represent means and STD from triplicates of one of the two independent experiments (**, P<0.001). (B) Comparison of sphere size distribution between LSChi; CD166hi and LSChi; CD166lo formed spheres. n.s., not significant. (C) Representative sphere images of LSChi;CD166hi and LSChi; CD166lo cells generated spheres. Scale bar: 100 µm. (TIF) [file pone.0042564.s001.tif]

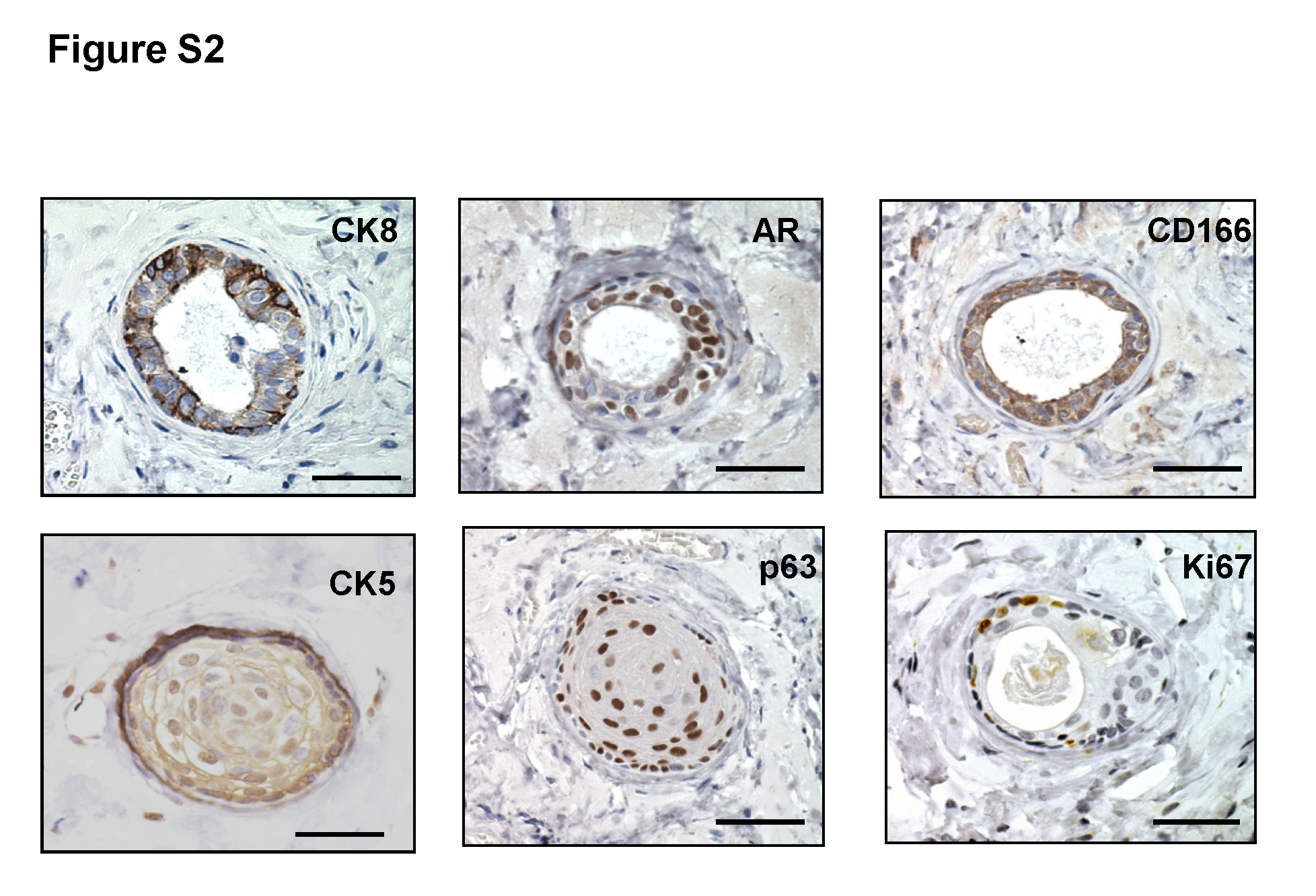

Supplement: Figure S2 — Immunohistochemical analysis of CD166hi human prostate epithelium-derived graft demonstrates nuclear expression of AR and p63, CK5 and CK8 positive cells and Ki67 positive cells within tubule structure. Scale bar: 50 µm. (TIF) [file pone.0042564.s002.tif]

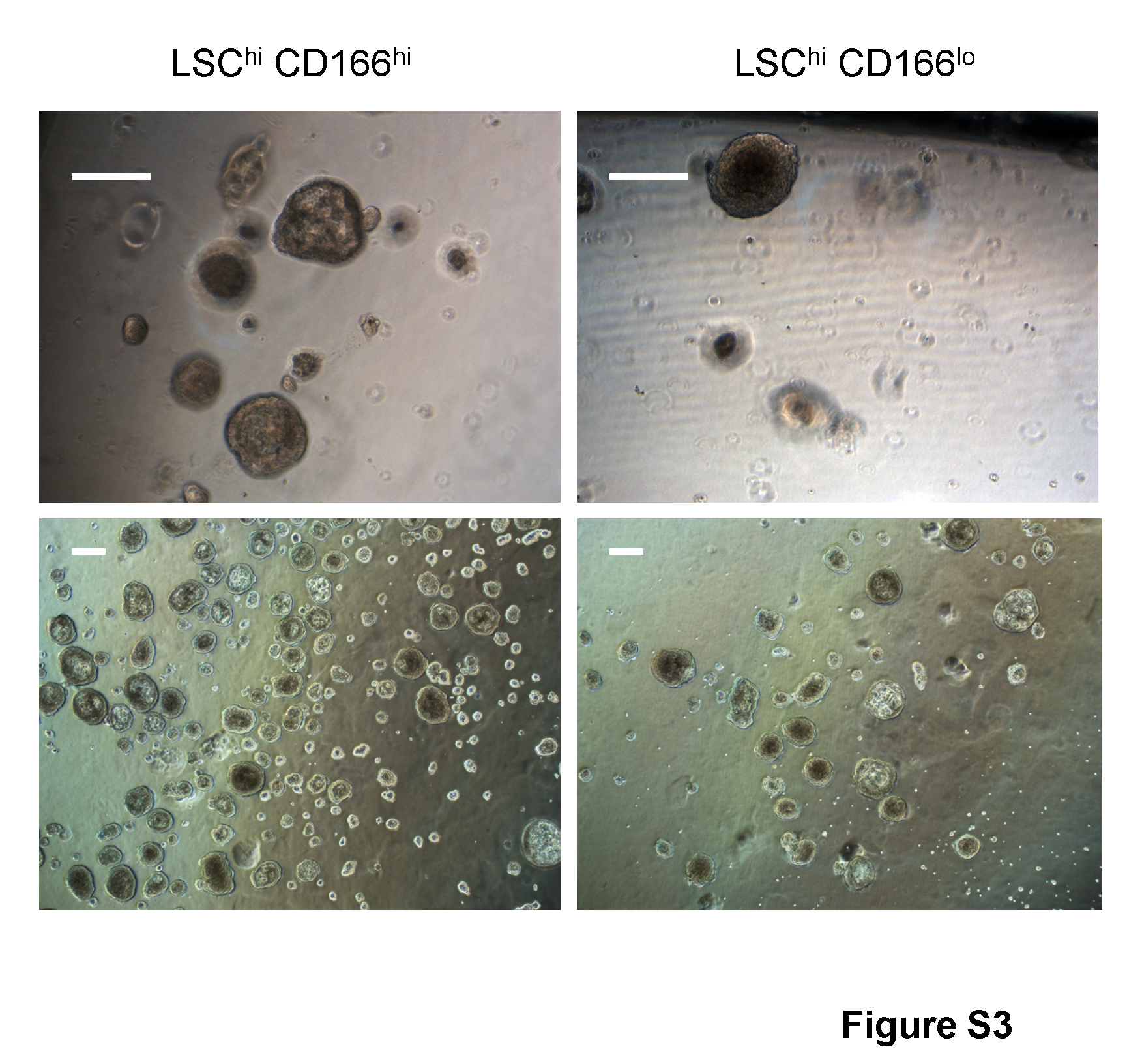

Supplement: Figure S3 — LSChi;CD166hi and LSChi; CD166lo cells isolated from Pten mutant prostate form spheres with similar size distribution. Representative sphere images of LSChi;CD166hi and LSChi; CD166lo cells generated spheres. Top: spheres maintained in matrigel. low: spheres released from matrigel after dispase treatment. Scale bar: 200 µm. (TIF) [file pone.0042564.s003.tif]

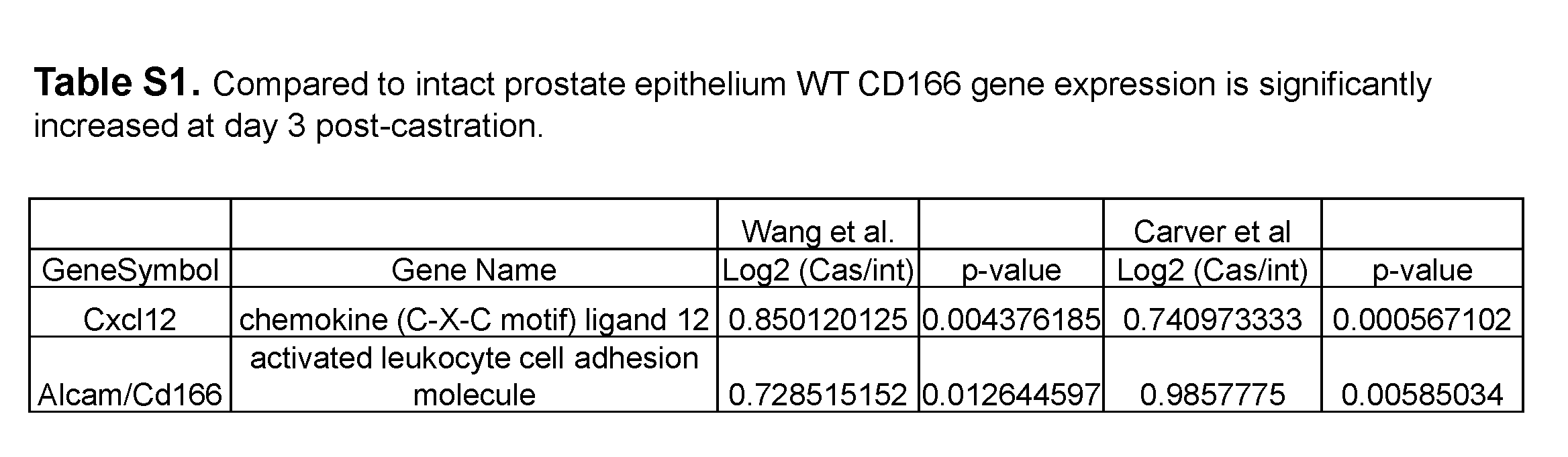

Supplement: Table S1 — Compared to intact prostate epithelium WT CD166 gene expression is significantly increased at day 3 post-castration. (TIF) [file pone.0042564.s004.tif]

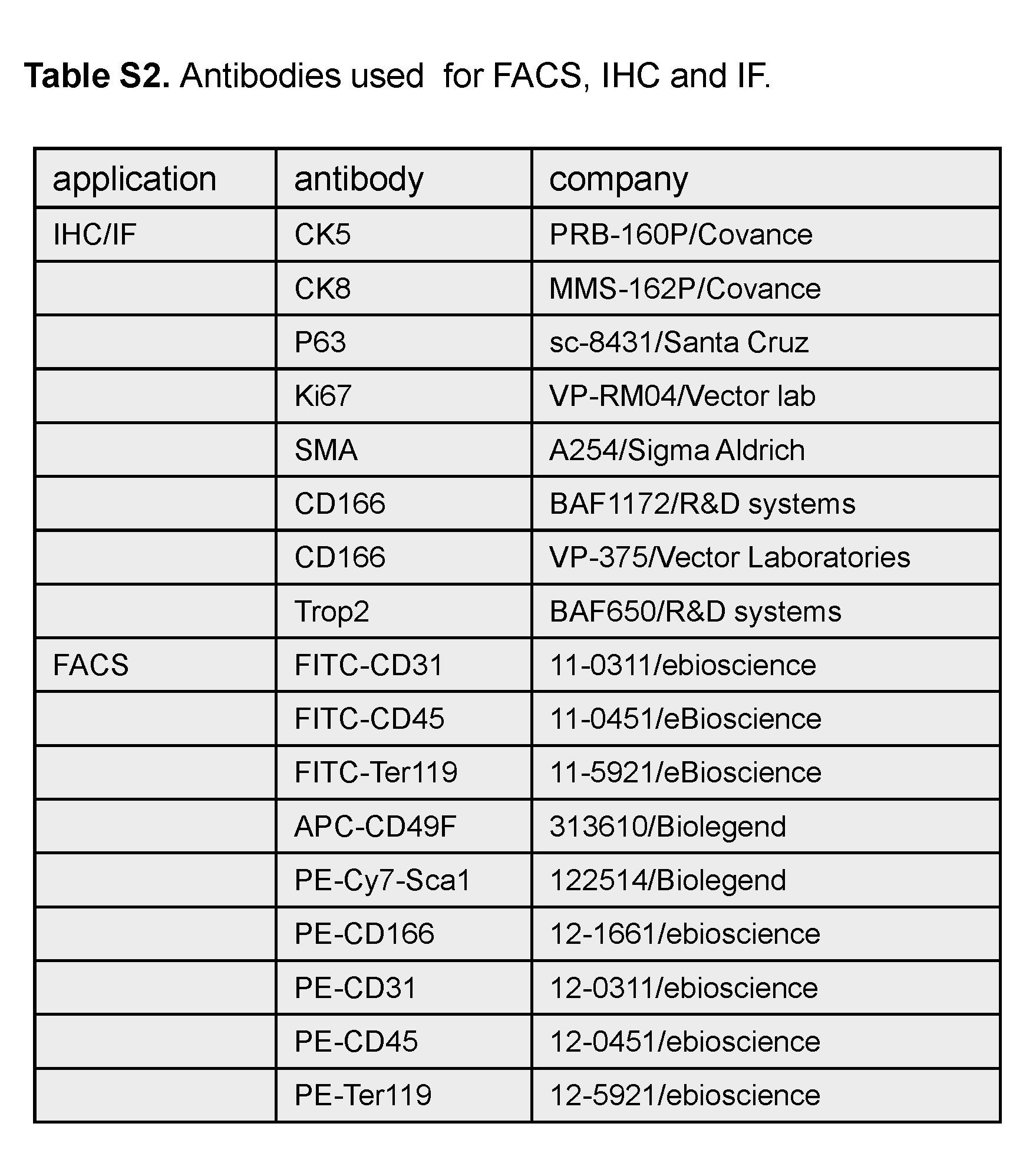

Supplement: Table S2 — Antibodies used for FACS, IHC and IF. (TIF) [file pone.0042564.s005.tif]
